# Parasacral transcutaneous electrical nerve stimulation in children with overactive bladder: comparison between sessions administered two and three times weekly

**DOI:** 10.1590/S1677-5538.IBJU.2020.0372

**Published:** 2021-03-05

**Authors:** Maria Luiza Veiga, Kaíse Oliveira, Vanessa Batista, Ananda Nacif, Ana Aparecida Martinelli Braga, Ubirajara Barroso

**Affiliations:** 1 Escola Bahiana de Medicina Departamento de Fisioterapia SalvadorBA Brasil Departamento de Fisioterapia, Escola Bahiana de Medicina, Salvador, BA, Brasil; 2 Escola Bahiana de Medicina e Saude Publica SalvadorBA Brasil Escola Bahiana de Medicina e Saude Publica Salvador, BA, Brasil; 3 Universidade Federal da Bahia Departamento de Urologia SalvadorBA Brasil Departamento de Urologia, Universidade Federal da Bahia – UFBA, Salvador, BA, Brasil

**Keywords:** Urinary Bladder, Overactive, Transcutaneous Electric Nerve Stimulation, Child

## Abstract

**Purpose:**

This study compares the results achieved following parasacral TENS administered using two different weekly schedules.

**Materials and Methods:**

Children of at least four years of age with a diagnosis of pure overactive bladder were included in this randomized clinical trial and treated with parasacral TENS (2 versus 3 sessions per week). All the participants also underwent standard urotherapy.

**Results:**

Sixteen children were included in the twice-weekly group and eighteen in the three times weekly group. There were no statistically significant differences between the two groups with respect to sex; however, there was a difference in age. There were no significant differences regarding complete resolution of urinary symptoms, with 8 children (50%) in the twice-weekly group and 11 children (61%) in the three times weekly group having their symptoms completely resolved (p=0.73). There was a significant difference in the DVSS score in both groups following TENS treatment compared to baseline (p=0.0001 for both groups), but not between groups. Evaluation of the bladder diary showed no difference between the groups before or after treatment.

**Conclusion:**

For children with overactive bladder who are unable to undergo parasacral TENS treatment three times weekly, the method can be administered successfully at twice-weekly sessions.

## INTRODUCTION

According to the International Children's Continence Society (ICCS), overactive bladder (OAB) is characterized by urinary urgency and may be associated with daytime incontinence, frequency and holding maneuvers used to avoid involuntary loss of urine (1). Urinary symptoms are present in 38% of children and consist of incontinence in 14% of cases and urgency in 18%, with daytime symptoms being more common in girls compared to boys (2). Lower urinary tract symptoms (LUTS) in children are associated with nocturnal enuresis, recurrent urinary tract infections (UTI) and constipation (1). LUTS are also associated with psychological alterations and social embarrassment. These issues tend to improve following successful treatment of urinary incontinence (3).

Parasacral transcutaneous electrical nerve stimulation (TENS) has been used to treat OAB in children since 2001 (4, 5). TENS was applied daily for 1-2 hours/day for periods exceeding six months. The success rate ranges from 13% to 86% depending on the form of stimulation and the intensity of the problem (6).

Although parasacral TENS has been shown to be effective in randomized clinical trials, the periodicity with which it should be performed has yet to be established. Various authors have described daily applications conducted at home (4, 5). In our clinic, parasacral TENS is administered on an outpatient basis three times a week for a total of twenty 20-minute sessions. In a randomized clinical trial, parasacral TENS was found to be associated with partial or complete response of symptoms in 94% of cases (62% and 38%, respectively), whereas only 31% of the sham group (TENS performed in the scapular region) had a similar outcome (7). Another long-term study conducted in our center using the same method showed that of the patients with urinary urgency or incontinence prior to treatment, 84% and 74%, respectively, remained asymptomatic for at least two years following treatment with parasacral TENS, with a recurrence rate of 10% in that study population (8).

Therefore, the effectiveness of this technique applied three times weekly has already been well documented. Nevertheless, this schedule is empirical and one involving fewer sessions could be beneficial both insofar as the convenience of the family is concerned and in reducing costs. The objective of the present study was to compare the results achieved following treatment involving different weekly schedules (twice weekly and three times weekly).

## MATERIALS AND METHODS

This randomized clinical trial included children of at least four years of age with a diagnosis of pure OAB who were treated with parasacral TENS. The internal review board of the Bahia School of Medicine and Public Health approved the study protocol under reference CAAE: 12141113.0.0000.5544 in compliance with the requirements of Resolution 466/12 of the Brazilian Ministry of Health. All the parents/guardians signed an informed consent form and the children signed an assent form.

Overactive bladder was defined as symptoms of urinary urgency with or without urge incontinence associated with a bell or tower shaped flow pattern at uroflowmetry and post-void residual urine at ultrasonography <10% of the expected bladder capacity for age (capacity in mL=age+1x30) or <20mL (1). Children with a urinary infection were treated prior to enrollment. TENS was the first treatment for urinary symptoms, with children who had previously been treated with anticholinergics or any other treatment being excluded from the study. Therefore, all the patients were treatment naïve. A pediatric urologist conducted the physical examinations, and children with urinary symptoms secondary to anatomical or neurological lower urinary tract abnormalities were excluded from the study.

Randomization was performed in blocks of four. Opaque envelopes were used to guarantee concealment of the study group allocation. An individual who was not involved in evaluating or treating the patients performed the entire procedure.

The treatment consisted of the application of an electric current with the use of surface electrodes placed symmetrically in the parasacral region (between S2 and S4), using a Dualpex Uro 961 device (Quark, Piracicaba, São Paulo, Brazil). The self-adhesive rubber electrodes used measured 5x5cm. A biphasic, symmetrical current with a frequency of 10Hz and pulse width of 700μs was used. Intensity was increased up to the level immediately below the motor point and sessions were conducted for 20 minutes on two or three non-consecutive days per week: Mondays, Wednesdays and Fridays in the case of the children treated three times weekly and Mondays and Fridays for those treated twice weekly.

All the participants in the study were submitted to urotherapy, which consisted of the following recommendations: to urinate at least every three hours; to avoid coffee, carbonated drinks, chocolate and citrus fruits during treatment; to urinate before going to bed; to drink more fluids during the day; and not to put off urinating when experiencing urgency. An illustrated booklet adapted for children and containing the aforementioned guidance was given to each participant.

Immediately after completion of the 20-session treatment, a professional who had not taken part in the treatment and who was unaware of the group to which the participant had been allocated evaluated the patients to analyze their response to treatment. If constipation was a complaint, the children were instructed to eat fiber-rich food and were referred to a specialist. The Rome III questionnaire (9) was also used to evaluate constipation and a two-day bladder diary (as standardized by the ICCS) was used prior to and following treatment to evaluate mean voiding frequency, as well as the mean and maximum urinary volume voided.

After the 20th session of treatment, symptoms were evaluated in three ways: using a visual analogue scale (VAS), by applying a structured questionnaire on the presence of lower urinary tract symptoms (urgency, daytime incontinence, frequent urination) and using the Dysfunctional Voiding Score System (DVSS). The version of the DVSS used has been validated for use in the Portuguese language (10). In brief, this instrument is used to quantify urinary symptoms and also includes two questions related to constipation and one on a history of stressful events. Scores above 9 for boys and above 6 for girls are considered abnormal. The VAS consists of a scale in which the patients and their parents/guardians record the degree of improvement experienced following treatment, with 0 reflecting no improvement and 10 complete response to treatment (100% improvement).

The Statistical Package for the Social Sciences (SPSS), version 17.0 for Windows, was used to create the database and to perform the descriptive and inferential statistics. The categorical variables are expressed as absolute and relative values (n; %) and the continuous variables with normal distribution as means±standard deviations (SD) of the mean. The inferences with respect to the categorical variables were made using the chi-square test and McNemar's test for paired samples. Student's t-test and the Wilcoxon test were used to compare the independent data. Significance was defined as p <0.05.

The groups were evaluated prior to treatment with respect to the frequency of urinary symptoms, sociodemographic data, DVSS score and data from the two-day bladder diary. After treatment, clinical response was evaluated within and between groups based on a reduction in clinical symptoms, DVSS score and data from the bladder diary. The VAS was used to compare the percentage of individuals who experienced complete response to treatment between the groups.

## RESULTS

Sixteen children were included in the twice-weekly group and eighteen in the three times weekly group. There were no statistically significant differences between the two groups with respect to sex, with 68.8% of the twice-weekly group consisting of girls compared to 61.1% of the three times weekly group. However, there was a difference in age, with a mean age of 6.44±2.12 years for the children in the twice-weekly group compared to a mean of 8.44±2.93 years in the three times weekly group (p=0.03).

There were no statistically significant differences regarding complete resolution of urinary symptoms, as evaluated by the VAS, with 8 children (50%) in the twice-weekly group and 11 children (61%) in the three times weekly group having their symptoms completely resolved (p=0.73). There was a significant difference in the DVSS score in both groups following TENS treatment compared to baseline. In the twice-weekly group, the mean baseline score of 11.14±3.2 decreased to a mean of 4.57±4.3 after treatment (p=0.0001) and in the three times weekly group, the mean score of 10.5±4.65 prior to TENS treatment decreased to 3.18±3.18 after treatment (p=0.0001). There was no statistically significant difference in DVSS score between the two groups following treatment (5.07±4.57 versus 3.11±3.10; p=0.17).

Evaluation of the urinary symptoms prior to and following treatment in each group, as well as an inter-group evaluation, is shown in Table-1. There was no statistically significant difference between the two groups following treatment.

**Table 1:** Comparison of urinary symptoms prior to and following parasacral transcutaneous electrical nerve stimulation administered two or three times weekly.

	Twice a Week (n = 16)				Three times a Week (n = 18)			Twice versus Three-times weekly
	Before	After	p-value*		Before	After	p-value*	p-value**
**Urgency (16)**	16 (100%)	6 (37.5%)		**Urgency (17)**	17 (100%)	5 (29.4%)		0.72
**Urge incontinence (16)**	14 (87.5%)	7 (43.8%)	0.039	**Urge incontinence (18)**	16 (89%)	5 (27.8%)	0.001	0.47
**Urinary incontinence (14)**	12 (85.7%)	8 (57%)	0.219	**Urinary incontinence (16)**	15 (94%)	5 (31.3%)	0.002	0.26
**Enuresis (16)**	12 (75%)	8 (50%)	0.289	**Enuresis (18)**	9 (50%)	7 (39%)	0.5	0.73
**Holding maneuvers (16)**	12 (75%)	5 (31.3%)	0.016	**Holding maneuvers (18)**	17 (94.4%)	4 (22.2%)	0.000	0.7
**Constipation (15)**	**7 (46.7%)**	**5 (33.3%)**	**0.688**	**Constipation (18)**	**8 (44.4%)**	**4 (22.2%)**	**0.289**	**0.69**

The data presented in this table were taken from the structured questionnaire used to evaluate urinary symptoms and from the Rome III criteria for the evaluation of constipation. In the case of urgency, the p-value is missing since this was one of the inclusion criteria, and therefore, a symptom experienced by all participants.

* McNemar's test.

** Chi-square test.

Evaluation of the bladder diary showed no difference in mean urinary frequency (p=0.87), mean urine volume voided (p=0.5) or maximum urine volume voided (p=0.67) between the two groups (Table-2).

**Table 2:** Bladder diary data prior to and following treatment as compared between the two and three-times weekly groups.

	Twice a week		Three times a week		p-value
	Before	After	Before	After	
Urinary frequency	10.5 ± 3.7	6.7 ±1	7.2 ± 0.7	7.1 ± 0.6	0.87
Mean volume of urine voided (mL)	97.4 ± 18.2	132.5 ± 23.7	117 ± 13.4	126.7 ± 16.9	0.50
Maximum volume of urine voided (mL)	186.2 ± 34.7	241 ± 47.7	248.6 ± 43	233.6 ± 21.6	0.67

## DISCUSSION

Parasacral transcutaneous electrical nerve stimulation has yielded good results for the treatment of bladder dysfunction in children when administered three times a week (7). De Paula et al. (11) reported that it is possible to administer treatment even once a week; however, the number of patients evaluated in that study was very small. Other previous protocols have been presented, but with the disadvantage that the treatment was more prolonged, although that disadvantage was offset by the comfort of being able to administer treatment at home, thereby reducing costs (4, 5). One of the advantages of outpatient treatment is that it is performed by a professional who is then able to reinforce the recommendations given during standard urotherapy. In addition, there is the theoretical advantage of being able to achieve greater current intensity levels when a physiotherapist is administering treatment compared to when the patient's parents are doing so; however, no studies have yet been conducted to compare the two methods. In this country, parents are not reimbursed for the costs of purchasing the device to use at home. On the other hand, the state Department of Health or the health insurance companies pay for outpatient treatment, making this form of therapy less expensive for patients.

Going to the clinic for treatment three times a week involves indirect costs that cannot be disregarded. The costs of transportation, food and having to miss work or school also have to be taken into account. Therefore, an attempt to reduce the number of weekly sessions is fully justified. The results of the present study show that it is possible to administer this treatment twice a week, since there was a significant reduction in lower urinary tract symptoms in both groups. There was also a reduction in enuresis and in constipation; however, this difference was not statistically significant, probably because few patients were suffering from constipation and enuresis prior to treatment.

One study, conducted in an attempt to reduce the number of sessions, evaluated patient response to parasacral TENS administered once a week in association with urotherapy and compared those patients to a control group submitted to placebo electrical stimulation in the scapular area plus urotherapy (11). The patients were submitted to 20 treatment sessions. At the end of the 20th session, there was no statistically significant difference between the groups with respect to the symptoms of urinary urgency and daytime incontinence. Sixty days after treatment, urinary urgency was found to have improved significantly only in the parasacral TENS group. Although those data appear to show that some patients do respond to once weekly sessions of parasacral TENS, it is impossible to know whether they would have improved faster and to a greater extent if they had been submitted to a greater number of sessions.

Other studies have published results on twice-weekly TENS treatment. One study conducted in Brazil treated 25 children with symptoms of urgency and urge incontinence using a protocol similar to that used in the present study (12). There was no difference in symptoms of urgency following TENS treatment; however, there was an improvement in the symptoms of urge incontinence. No comparison was made with any other treatment group. Another randomized controlled study compared sacral TENS in association with interferential current and urotherapy with urotherapy alone in 36 children. Children diagnosed with bladder emptying problems (infrequent voiding, straining at urination, post-void residual urine and an interrupted pattern on uroflowmetry) were included in the study. The results were favorable, with an increase in urinary frequency and a reduction in bladder volume (13). The sample included and the treatment protocol both differed in that study from those of the present study. In the present study, both groups experienced an improvement in symptoms of urgency and urge incontinence. Nevertheless, despite a reduction in the percentage of children experiencing incontinence following treatment in the twice-weekly group, this difference was not statistically significant. Explanations for this may include a different response to treatment in the two groups or a type 2 error due to a small sample size.

Briefly, TENS has been found to play a significant role in the improvement of urodynamic patterns and the objective symptoms of OAB in children with neurogenic and non-neurogenic dysfunctions (14-16) using differing numbers of weekly sessions. The search for safe and effective treatment resources may benefit patients and their families. A recent publication described a pilot study conducted to evaluate a new treatment option, percutaneous electrical stimulation (PENS) applied in the sacral region (17). Further studies are currently being conducted.

The limitations associated with the present study include the small sample size of patients. For almost all the symptoms, results were better when parasacral TENS was administered three times weekly; however, this did not translate into statistical significance. Although there could be a type 2 error, the present data show that from the clinical point of view and in accordance with the results presented here, nerve modulation can be administered on a twice weekly basis when parents are unable to attend the clinic three times a week.

The symptoms were evaluated subjectively; therefore, there could be a certain degree of imprecision. Since it is impossible to blind the therapist, it is always possible that there could be some effect of this bias in the results. In the present study, this is minimized by randomization and by blinding the final evaluator. Furthermore, since all the patients were submitted to standard urotherapy, the results may have been improved by the addition of this treatment. Nevertheless, parasacral TENS is routinely administered together with standard urotherapy and the idea of the present study was to replicate the usual clinical setting. It is possible that the urinary symptoms of some patients could have been resolved if constipation had been treated first. However, part of our investigation project includes a research protocol in which TENS is evaluated as the first treatment for constipation. We believe that in view of the high failure rate with the usual treatment methods and the high recurrence rate, TENS may play a relevant role in the treatment of functional constipation in children.

## CONCLUSION

The present study suggests that the results achieved with TENS administered twice weekly may be similar to those achieved when TENS is administered three times weekly for the treatment of symptoms of OAB in children.
